# Counterdiabatic ADAPT-VQE for Molecular Simulation

**DOI:** 10.1021/acs.jctc.6c00197

**Published:** 2026-06-15

**Authors:** Diego Tancara, Herbert Díaz-Moraga, Dardo Goyeneche

**Affiliations:** Facultad de Física, 28033 Pontificia Universidad Católica de Chile, Santiago 7820436, Chile

## Abstract

Among the variational quantum algorithms designed for NISQ devices, the adaptive derivative-assembled problem-tailored variational quantum eigensolver (ADAPT-VQE) stands out for its robustness against barren plateaus, particularly in estimating molecular ground states. On the other hand, counterdiabatic algorithms have shown advantages in both performance and circuit depth when compared to standard adiabatic approaches. In this study, we propose a hybrid method that integrates the ADAPT-VQE framework with counterdiabatic driving within an adiabatic evolution scheme. Specifically, we map the molecular Hamiltonian to a qubit representation and construct an adiabatic Hamiltonian, from which an approximate adiabatic gauge potential is computed using nested commutators. The resulting operator terms define the operator pool, and the ADAPT-VQE algorithm is applied to iteratively select the most relevant elements for the ansatz. Our results demonstrate improvements in performance and reductions in circuit depth compared to using either counterdiabatic algorithms or ADAPT-VQE with fermionic excitation operators, thus supporting the effectiveness of combining both paradigms in molecular simulations.

## Graphical abstract

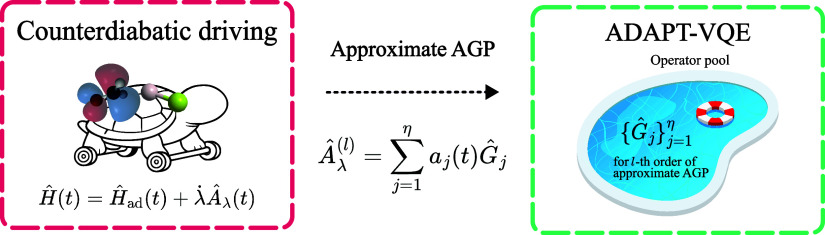

## 1 Introduction

Research on quantum computing has expanded significantly over the last few decades, with several experimental demonstrations of quantum advantage emerging even under the constraints of current noisy intermediate-scale quantum (NISQ) devices.^1–9^ This has motivated the development of quantum algorithms specifically designed for the NISQ era, which has become an active area of research.^10^ In this context, the variational quantum eigensolver (VQE) has emerged as a promising alternative, combining a shallow quantum circuit built from a chosen ansatz with classical optimization to approximate ground states efficiently on NISQ devices. In the foundational work,^11^ an ansatz for molecular simulations was proposed based on the unitary coupled cluster with single and double excitations (UCCSD). This approach involves the exponential of a linear combination of Fermionic excitation operators, where the variational parameters correspond to the weights assigned to each excitation term. Another notable approach applied to molecular simulations is the hardware-efficient ansatz,^12^ in which local operations are implemented using standard quantum gates, and entanglement between qubits is generated through direct hardware-level interactions.

Across the different ansatzes employed in VQE and variational quantum algorithms in general, the barren plateau problem has emerged as a serious challenge and has been extensively investigated in recent years.^13,14^ This problem is reflected in the exponential suppression of the cost-function gradient variance as the system size increases, leading to severe training difficulties for variational quantum algorithms when scaling to larger numbers of qubits. This can be characterized by the exponential vanishing variance of the cost function gradients regarding the system size, which makes variational quantum algorithms increasingly difficult to train as the number of qubits grows. Consequently, designing algorithms that incorporate reliable mechanisms to alleviate barren plateaus continues to be an active and relevant line of research. Among the limited set of variational quantum algorithms that are robust to barren plateaus, adaptive derivative-assembled problem-tailored VQE (ADAPT-VQE) stands out as a notable example,^15^ with its principal characteristic being the dynamic construction of the ansatz. This robustness stems from two key mechanisms. First, ADAPT-VQE employs a warm-start strategy in which the parameters optimized at each iteration are recycled as the initial point for the next, preventing the optimization from restarting in randomly initialized regions of the parameter landscape. Second, new operators are incorporated into the ansatz from a predefined operator pool based on a gradient criterion, selecting at each step the operator that produces the largest energy gradient. Together, parameter recycling and gradient-guided ansatz growth keep the optimization localized in regions with measurable gradients, thereby avoiding local minima and flat regions that characterize barren plateaus.^15^

Initially, ADAPT-VQE was introduced for molecular simulations, where the operator pool is composed of single and double excitations operators mapped to the qubit operators. These operators, which correspond to those used in the exponential form of the UCCSD ansatz, generate the operator pool. By adaptively selecting only the most relevant components, the algorithm achieves higher accuracy and leads to significantly shallower circuits, ultimately outperforming standard UCCSD ansatz.^16^ Subsequently, alternative operator pools were introduced, in which the Fermionic single and double excitations are replaced by their qubit operators representation through direct Pauli strings,^17^ leading to an improvement in circuit depth compared with the original ADAPT-VQE formulation. Moreover, incorporating qubit excitations directly, rather than relying on Fermionic excitations mapped to the qubit representation,^18^ and including double qubit excitations within the same spin–orbitals,^19^ has led to improved circuit depth and enhanced accuracy.

The ADAPT-VQE has also been applied to problems in other contexts. For instance, an alternative version of the algorithm was employed to prepare the 100-qubit vacuum state of the Schwinger model using a superconducting qubit quantum computer.^20^ A modified formulation of ADAPT-VQE was also proposed for quantum dynamics simulation, incorporating McLachlan’s variational principle in the cost function.^21^ These results highlight the versatility of the ADAPT-VQE strategy and have motivated further developments, including improved measurement protocols and alternative adaptive schemes.^22,23^

Another approach for ground state preparation is digitized counterdiabatic quantum optimization (DCQO).^24^ This method can be understood as a digitized implementation of adiabatic quantum computing^25^ enhanced with counterdiabatic driving that is introduced to suppress nonadiabatic transitions during the evolution. In contrast to standard adiabatic protocols, which require long evolution times to follow the adiabatic theorem, counterdiabatic driving enables the system to closely follow the instantaneous ground state even for finite-time evolutions. The digitization of the protocol through Trotterization of the time-evolution operator makes DCQO compatible with gate-based quantum hardware, enabling the preparation of ground states of optimization and many-body Hamiltonians with reduced circuit depth compared to digitized adiabatic evolution.^24^ The DCQO has been applied to different problems like protein folding,^26^ logistic scheduling,^27^ portfolio optimization^28^ and recently to molecular simulation.^29^

In this work, we propose a hybrid quantum–classical algorithm that combines the ADAPT-VQE strategy for operator selection and ansatz construction with concepts from counterdiabatic quantum computing to approximate ground states. Focusing on the shorter evolution times, we retain only the counterdiabatic driving terms and use them to define an operator pool for the ADAPT-VQE implementation. This approach is applied to the ground state estimation of molecular Hamiltonians.

## 2 Methods

In this section, we outline the construction of the adiabatic Hamiltonian starting from a molecular Hamiltonian, review the standard counterdiabatic protocol, and finally present our algorithm.

### 2.1 Molecular Hamiltonian to Adiabatic Hamiltonian

The determination of molecular ground states and energies is fundamentally governed by the electronic structure which corresponds to the Coulomb interactions between electrons and nuclei. In the low-energy regime, the Born–Oppenheimer approximation^30^ is well-justified by the fact that nuclei are significantly heavier than electrons. This approximation treats nuclei as stationary point charges, effectively decoupling nuclear and electronic motion. Within this approximation, the problem reduces to solving the time-independent Schrödinger equation for the electronic Hamiltonian, *H*_f_, given by

$$H_{f}=-\frac{1}{2}\sum_{i}\nabla_{r_{i}}^{2}-\sum_{i}\sum_{j}\frac{Z_{j}}{\left|R_{j}-r_{i}\right|}+\sum_{i<j}\frac{1}{\left|r_{i}-r_{j}\right|}$$ 1

where **r**_*i*_ and **R**_*j*_ denote the positions of electrons and nuclei, respectively, and *Z*_*j*_ is the atomic number of the *j*-th nucleus. This Hamiltonian encapsulates the kinetic energy of the electrons, the Coulombic attraction between electrons and nuclei, and the interelectronic repulsion in atomic units (a.u.).

To encode this problem into a quantum computer, we follow the standard second quantization formalism, where the Hamiltonian is written in terms of Fermionic creation and annihilation operators acting on Fock space:

$${\hat{H}}_{f}=\sum_{ij}h_{ij}{\hat{a}}_{j}^{†}{\hat{a}}_{i}+\frac{1}{2}\sum_{ijkl}h_{ijkl}{\hat{a}}_{l}^{†}{\hat{a}}_{k}^{†}{\hat{a}}_{j}{\hat{a}}_{i}$$ 2

Here, the coefficients *h*_*ij*_ and *h*_*ijkl*_ are the one-body and two-body integrals, respectively, defined as

$$h_{ij}=\int \phi_{i}^{*}(r)\left(-\frac{1}{2}\nabla^{2}-\sum_{n}\frac{Z_{n}}{r_{n}}\right)\phi_{j}(r)dr$$ 3

$$h_{ijkl}=\iint \frac{\phi_{i}^{*}(r_{1})\phi_{j}^{*}(r_{2})\phi_{k}(r_{2})\phi_{l}(r_{1})}{\left|r_{1}-r_{2}\right|}dr_{1}dr_{2}$$ 4

Where the spin–orbital functions {ϕ_*k*_} are the set of basis in which the Fock space is represented. Chemical basis sets are utilized in order to approximately express the molecular orbitals of the molecules.

Finally, the isomorphism between the Fermionic algebra and the *n* qubit algebra $\mathrm{su}(2^{n})$ is established via mappings such as the Jordan-Wigner, Parity, or Bravyi-Kitaev transformations.^31^ This allows the Hamiltonian to be fully expressed in terms of Pauli strings:

$${\hat{H}}_{f}=\sum_{{\hat{P}}_{i}\in P}h_{i}{\hat{P}}_{i}$$ 5

where *h*_*i*_ is the coefficient associated with the *P*_*i*_ Pauli string acting on the space of *n* qubits, and $P=\{\hat{I},\hat{X},\hat{Y},\hat{Z}{\}}^{⊗n}$ the Pauli group associated with it.

Adiabatic quantum computing exploits the adiabatic theorem in order to compute the ground state of a target Hamiltonian, *Ĥ*_f_, by preparing a known ground state of an initial Hamiltonian *Ĥ*_*i*_ and by considering the evolution of a schedule function λ­(*t*) in the following adiabatic Hamiltonian:

$${\hat{H}}_{\mathrm{ad}}=(1-\lambda (t)){\hat{H}}_{i}+\lambda (t){\hat{H}}_{f}$$ 6

where the schedule function satisfies the boundary conditions λ(0) = 0 and λ­(*T*) = 1, where *T* is the final time. This construction ensures that the adiabatic Hamiltonian operators starts at the *Ĥ*_*i*_ and evolves such that ends in *Ĥ*_f_. While the final Hamiltonian is determined by the specific problem to be solved—in this case, the molecular Hamiltonian *H*_f_ expressed as a linear combination of Pauli strings^5^—the initial Hamiltonian *H*_*i*_ is selected for its efficiency in enabling easy ground-state preparation. As established in the literature,^30^ for molecular problems, the Hartree–Fock state |HF⟩ is a good initial reference state. In the second quantization framework, the Hartree–Fock state is defined as the single Slater determinant of spin–orbitals. We define the corresponding occupation vector as **o** = (*o*_*n*–1_, ···, *o*_0_), where *o*_*k*_ = 1 for the *N* lowest-energy spin–orbitals and *o*_*k*_ = 0 otherwise. Depending on the Fermion-to-qubit mapping utilized, this occupation vector is transformed into a specific computational basis state: |HF⟩ = |*b*_*n*–1_ *b*_*n*–2_, ···, *b*_0_⟩, where the bitstring {*b*_*k*_} is uniquely determined by the mapping and the number of electrons in the basis. To follow the adiabatic protocol, the system must be initialized in the ground state of the initial Hamiltonian *Ĥ*_*i*_. Therefore, we construct a local initial Hamiltonian *Ĥ*_*i*_ such that its ground state corresponds to the encoded Hartree–Fock state:

$${\hat{H}}_{i}=\sum_{k=0}^{n-1}(-1{)}^{b_{k}+1}{\hat{Z}}_{k}$$ 7

where ${\hat{Z}}_{k}≡I^{⊗n-k}⊗\hat{Z}⊗I^{⊗k-1}$ is the local *Ẑ* Pauli gate acting on the *k*-th qubit and I is the local identity operator. This initial Hamiltonian is chosen because it is easy to implement due to the locality of its operators and because its ground state corresponds to the Hartree–Fock state when this state is known. To illustrate this, we consider a simple case of four spin–orbitals represented by four qubits for a two-electron molecule. Following the ordering |α_1_α_0_β_1_β_0_⟩, where α_*i*_ (β_*i*_) represents the occupational state of the *i*–th spin up (down) spatial orbital, the Hartree–Fock state is |HF⟩ = |0101⟩. The initial Hamiltonian is then given by

$${\hat{H}}_{i}=-{\hat{Z}}_{0}+{\hat{Z}}_{1}-{\hat{Z}}_{2}+{\hat{Z}}_{3}$$ 8

where *Ẑ*|0⟩ = |0⟩ and *Ẑ*|1⟩ = −|1⟩. We can verify that |0101⟩ is the ground state with eigenvalue −4. Therefore, using eq 7 we can encode the *n*-qubit Hartree–Fock state as the ground state and construct the corresponding adiabatic Hamiltonian.

### 2.2 Counterdiabatic Protocol

Since adiabatic evolution must proceed slowly to satisfy the adiabatic theorem, its direct use can become impractical for quantum computing. To mitigate this limitation, one can incorporate an additional counterdiabatic driving term that accelerates the protocol and suppresses diabatic excitations. The total Hamiltonian with counterdiabatic driving is

$${\hat{H}}_{\mathrm{cd}}=(1-\lambda (t)){\hat{H}}_{i}+\lambda (t){\hat{H}}_{f}+\hat{\lambda }(t){\hat{A}}_{\lambda }$$ 9

where λ̇(0) = λ̇(*T*) = 0 and *Â*_λ_ is the adiabatic gauge potential (AGP). In practice, evaluating *Â*_λ_ requires access to the instantaneous eigenstates and eigenvalues of the adiabatic Hamiltonian *Ĥ*_ad_, which makes its direct computation impractical. However, we can calculate approximate versions of the AGP without the spectral information on *Ĥ*_ad_. One of these approximations is given in ref 32. and is based in an expansion of nested commutators. The corresponding *l*-th order approximation can then be written as

$${\hat{A}}_{\lambda }^{\left(l\right)}=i\sum_{k=1}^{l}\alpha_{k}(t){\hat{O}}_{2k-1}(t)$$ 10

where the nested commutators are

O^k=[H^ad,[H^ad,...[H^ad︸k,∂λH^ad]]] 11

and the coefficients α_*k*_(*t*) can be obtained by minimizing the action *S*_*l*_ = Tr­[*G*_*l*_^2^], with *G*_*l*_ = ∂_λ_ *Ĥ*_ad_ – *i*[*Ĥ*_ad_, *Â*_λ_^(*l*)^]. In the limit *l* → *∞*, the series reproduces the exact AGP. The counterdiabatic protocol has been used in the context of quantum computing with an approximate AGP, typically with *l* = 1, together with a Trotter-Suzuki decomposition to digitize the time-evolution operator generated by *Ĥ*_cd_, showing improvements in circuit depth and performance regarding the standard adiabatic protocol.^24^

### 2.3 Counterdiabatic ADAPT-VQE

The VQE has become one of the central algorithms in the NISQ era, particularly in quantum chemistry. However, its accuracy depends greatly on the choice of ansatz, which has motivated the development of many tailored constructions designed to balance expressiveness and circuit depth. With the parametrized ansatz, the normalized state |ψ­(θ)⟩ is used in a classical optimization to minimize the energy, which is given by

$$E=\min_{\theta }\langle \psi (\theta )|{\hat{H}}_{f}|\psi (\theta )\rangle$$ 12

Common fixed ansatz for molecular simulations are based on excitation operators, which often lead to large circuit depths that are susceptible to barren plateaus. In this context, ADAPT-VQE algorithm was introduced to address these issues by building the ansatz iteratively from energy gradient criterion, yielding more compact circuits and showing improved robustness against barren plateaus, thus enhancing its potential for near-term quantum applications.

The ADAPT-VQE algorithm begins by defining an operator pool consisting of a set of operators { ${\hat{V}}_{k}$ }_*k* = 1_^*M*^, where the original choice for ${\hat{V}}_{k}$ corresponds to the single and double excitation operators mapped to the qubit representation. Their Pauli decomposition leads to the Pauli strings used in more recent formulations, so that in general ${\hat{V}}_{k}$ ∈ {*X̂*, *Ŷ*, *Ẑ*, *Î*}^⊗*n*^. The algorithm is initialized with a reference state |ψ_*i*_⟩ at iteration *j* = 0, usually the Hartree–Fock state. For each operator ${\hat{V}}_{k}$ in the pool, the energy gradient with respect to a variational parameter θ_*k*_ is evaluated when applying the unitary *e* ^–*i*θ_*kV̂k*_^ to the current ansatz state |ψ_*j*_⟩, at θ_*k*_ = 0. This derivative takes the form of a commutator expectation value:

$$g_{k}=\frac{\partial E}{\partial \theta_{k}}{|}_{\theta_{k}=0}=i\langle \psi_{j}|[{\hat{V}}_{k},\hat{H}]|\psi_{j}\rangle$$ 13

and the collection of all such values defines the gradient vector $\vec{g}=(g_{1},...,g_{M})$ . If the $l_{2}$ -norm $∥\vec{g}{∥}_{2}$ is less than a predefined threshold ϵ, the algorithm terminates. Otherwise, the operator *V*_*k*_ corresponding to the maximum absolute component of $\vec{g}$ is selected (denoted *V* ^max^) and added to the ansatz. The ansatz is then updated as

$$\left|\psi_{j+1}\rangle =e^{-i\theta {\hat{V}}^{\max}}|\psi_{j}\right\rangle$$ 14

where θ is a newly introduced variational parameter. A VQE optimization is then performed to update all parameters to the optimum value and obtain the new optimal state |ψ_*j*+1_^opt^⟩. This state is then set as the input for the next iteration: |ψ_*j*+1_^opt^⟩ → |ψ_*j*_⟩ and the process repeats until $∥\vec{g}∥<\epsilon$ .

The choice of an appropriate operator pool is a crucial step in the ADAPT-VQE algorithm. Interestingly, previous results in adaptive version of Quantum Approximate Optimization Algorithm^33^ have shown that the operators selected during the first iterations exhibit a strong overlap with the dominant terms of an approximate counterdiabatic Hamiltonian. This observation suggests that counterdiabatic evolution is not only relevant for understanding the dynamics of adaptive ansatz, but can also serve as a physically motivated principle for designing the operator pool. In this work we therefore construct the operator pool from the approximate AGP as follows. The counterdiabatic evolution can be analyzed in different regimes determined by the total evolution time *T*. For sufficiently short final times, the Hamiltonian varies rapidly, making the counterdiabatic contribution more relevant than the adiabatic term. This situation is commonly referred to as the impulse regime,^28^ in which the dynamics is dominated by the rate of change of the schedule function and satisfies the condition |λ­(*t*)| ≪ |λ̇(*t*)|. Under this assumption, the Hamiltonian can be approximated by

$${\hat{H}}_{\mathrm{cd}}\approx \dot{\lambda }(t){\hat{A}}_{\lambda }^{\left(l\right)}$$ 15

Once performed the mapping that transforms *Ĥ*_cd_ to the Pauli set, the *l*-th order approximated AGP can be writen in terms of generators or Pauli strings by solving the nested commutators:

$${\hat{A}}_{\lambda }^{\left(l\right)}=\sum_{j=1}^{\eta }a_{j}(t){\hat{G}}_{j}$$ 16

where η is the number of Pauli strings of *Â*_λ_^(*l*)^ and the coefficients *a*_*i*_(*t*) are weights associated with the *j*-th Pauli strings *Ĝ*_*j*_, that are obtained by the ponderation of the terms α_*k*_(*t*), and combinations of powers of λ­(*t*) and (1 – λ­(*t*)) arising from the nested commutators. The operator pool is then constructed by fixing the *l*-th order giving rise to the operator pool {*Ĝ*_*j*_}_*j* = 1_^η^.

This operator pool is motivated by the dynamics of the counterdiabatic protocol, and in particular by the Trotterized time-evolution operator generated by the counterdiabatic Hamiltonian in eq 15, which takes the form:

$$\hat{U}(T)=\prod_{k=1}^{p}\prod_{j=1}^{\eta }\exp[-i\dot{\lambda }(k\delta t)a_{j}(k\delta t)\delta t{\hat{G}}_{j}]$$ 17

The expression above has been implemented on quantum processors as a digitized counterdiabatic quantum algorithm, where in practice only the case *l* = 1 is used, since η increases significantly with the *l*-th AGP approximation, increasing the circuit depth. Related works have also used the time-evolution operator of the counterdiabatic protocol as inspiration to construct a variational ansatz, replacing the time-dependent functions by variational parameters associated with the operators *Ĝ*_*j*_. In that setting, the ansatz takes the form:

$$\hat{U}(\theta )=e^{-i\sum_{j}^{\eta }\theta_{j}{\hat{G}}_{j}}$$ 18

This approach has also been applied to molecular simulations.^29^ However, the same issue of a rapidly increasing η appears for higher *l*-th AGP approximations. In this work, we instead propose using the full set {*G*_*j*_}_*j* = 1_^η^ as the operator pool, and employing ADAPT-VQE to select the most relevant terms. This strategy enables the use of *l* > 1, since increasing *l* only enlarges the operator pool rather than the circuit depth of the ansatz, so that only the most relevant contributions from higher-order AGP approximations are incorporated into the ansatz. It is important to note that our algorithm retains the key structural elements that are known to mitigate barren plateau effects and local minimum in ADAPT-VQE. In particular, the optimization proceeds iteratively using the parameters obtained in the previous step as a warm start and operator inclusion follows the same gradient-based selection principle, whereby the operator producing the largest energy gradient is appended at each iteration. We refer to this algorithm as CD-ADAPT from here on, and define it as follows:
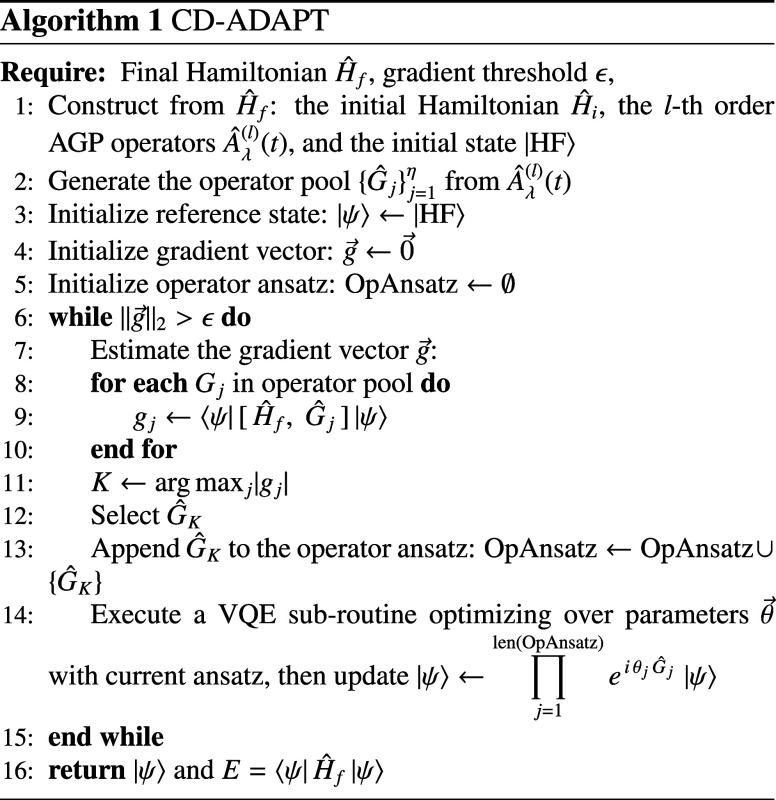


## 3 Results

In this section we present the main results obtained with the CD-ADAPT algorithm. We first examine the size of the operator pool generated by our construction and the effect of the approximations involved. We then report the energies obtained for three molecular systems. Finally, we benchmark the performance of CD-ADAPT against ADAPT-VQE, digitized counterdiabatic quantum algorithms, and other ADAPT-VQE variants.

### 3.1 Operator Pool Size

In our algorithm, the size of the operator pool depends strongly on the specific final Hamiltonian *Ĥ*_f_, since the operators {*G*_*j*_}_*i* = 1_^η^ arise from nested commutators involving both *Ĥ*_*i*_ and *Ĥ*_f_. Therefore, the choice of molecular basis sets and mapping to qubit operators determines in the operator pool in a more relevant way than standard Fermionic excitation operator pools, since these mappings constrain to a certain subset of the *n*-qubit Pauli group that we will use. An important aspect of our AGP-based pool is the structure of antisymmetric operators, which can be understood as follows. The Hamiltonian *Ĥ*_f_ is real by construction, as it originates from Fermionic operators, a property that is preserved under qubit mappings. Therefore, *Ĥ*_f_ is real and Hermitian, implying that it is symmetric and that all its Pauli strings contain an even number of *Y* operators.

By considering the case *l* = 1, the commutator of *Ĥ*_f_ with *Ĥ*_*i*_ = ∑_*k*_ *Z*_*k*_, which consists only of local *Z*_*k*_ operators, can be understood by analyzing its action on a local site *k* within a Pauli string of *Ĥ*_f_. Each term in the commutator reduces to evaluating [*Z*_*k*_, *P*], where *P* is a Pauli string appearing in *Ĥ*_f_.

If the operator acting on site *k* in *P* is either *I* or *Z*, the commutator vanishes. However, if the local operator is *X* or *Y*, the commutator generates a nonzero contribution:

$$\begin{array}{cc} {}[Z_{k},X_{k}]=2iY_{k}, & [Z_{k},Y_{k}]=-2iX_{k} \end{array}$$

Thus, the action of the commutator effectively flips the local Pauli operator between *X* and *Y*, while introducing a factor of *i*. As a consequence, each resulting Pauli string acquires an odd number of *Y* operators, in contrast to the even-parity structure of *Ĥ*_f_. The same argument extends to higher *l*-th orders, as the nested commutator is applied an odd number of times.

This parity change implies that the operators generated through the commutator are purely imaginary and antisymmetric. This property is central to the construction of the AGP-based pool. The restriction to operators with odd *Y*-parity has been identified in previous works^17^ and constitutes a necessary condition for obtaining nonvanishing contributions in the commutator relevant for gradient evaluation.

An advantage of our algorithm is that we can increase the precision by obtaining a more precise operator pool by choosing the *l*-th order approximation of AGP (Table 1). For example for *l* = 1 we have

$${\hat{A}}_{\lambda }^{l=1}=i\alpha_{1}(t)[{\hat{H}}_{i},{\hat{H}}_{f}]$$ 19

and the Pauli strings *Ĝ*_*j*_:

$${\hat{G}}_{j}\in \{[{\hat{H}}_{i},{\hat{H}}_{f}]\}$$ 20

are obtained by computing the commutator using the algebra of the Pauli Group. For *l* = 2 the expression is more complex but it is possible to obtain by simple rules of commutators:

$${\hat{A}}_{\lambda }^{l=2}=i\alpha_{1}(t)[{\hat{H}}_{i},{\hat{H}}_{f}]+i\alpha_{2}(t)((1-\lambda^{2}(t)[{\hat{H}}_{i},[{\hat{H}}_{i},[{\hat{H}}_{i},{\hat{H}}_{f}]]]+\lambda (t)(1-\lambda (t))([{\hat{H}}_{i},[{\hat{H}}_{f},[{\hat{H}}_{i},{\hat{H}}_{f}]]]+[{\hat{H}}_{f},[{\hat{H}}_{i},[{\hat{H}}_{i},{\hat{H}}_{f}]]])+\lambda^{2}(t)[{\hat{H}}_{f},[{\hat{H}}_{f},[{\hat{H}}_{i},{\hat{H}}_{f}]]])$$ 21

and the Pauli strings *Ĝ*_*j*_ are the operators that appear when evaluating these commutators:

$${\hat{G}}_{j}\in \{[{\hat{H}}_{i},{\hat{H}}_{f}],[{\hat{H}}_{i},[{\hat{H}}_{i},[{\hat{H}}_{i},{\hat{H}}_{f}]]],[{\hat{H}}_{f},[{\hat{H}}_{i},[{\hat{H}}_{i},{\hat{H}}_{f}]]],[{\hat{H}}_{f},[{\hat{H}}_{i},[{\hat{H}}_{i},{\hat{H}}_{f}]]],[{\hat{H}}_{f},[{\hat{H}}_{f},[{\hat{H}}_{i},{\hat{H}}_{f}]]]\}$$ 22

where we observe a substantial increase in the number of operators in the pool for *l* = 2. We use the tensorized Pauli decomposition (TPD) algorithm^36^ to extract the Pauli strings from the approximate AGP. As an illustration, for the molecules lithium hydride (LiH), hydrogen fluoride (HF), and linear beryllium hydride (BeH_2_), and using a 10-qubit model, the corresponding sizes of the operator pool are

**Table 1:** Operator Pool Size η for *l* = 1 and *l* = 2

	*N*° operators (*l* = 1)	*N*° operators (*l* = 2)
LiH	216	9148
HF	288	19,540
BeH_2_	108	4712

The growth of η from *l* = 1 to *l* = 2 is substantial, and one might expect a continued increase with higher *l*-th orders. However, the exact AGP provides an upper bound on the number of Pauli strings that can be generated in its approximate AGP. Figure 1a shows numerical results utilizing the TPD algorithm for the size of the operator pool at different orders *l* for the LiH, HF and BeH_2_ molecules, where we observe that the sequence converges toward a constant value. We emphasize that, beyond the pool size, the measurement cost of the operator-selection step is also determined by how efficiently the Pauli strings involved in the gradient evaluations can be grouped into mutually commuting sets. Such grouping strategies can significantly reduce the number of measurement settings required in ADAPT-VQE,^34^ and they can in principle be incorporated into our AGP-based pool as well. At the same time, other strategies can also be considered to reduce the required resources. For instance, one may incorporate a penalty for operators associated with larger circuit depth on a specific hardware architecture into the gradient-based selection criterion, as has been proposed recently.^35^ This strategy can likewise be applied to our AGP-based pool.

**Figure 1:**
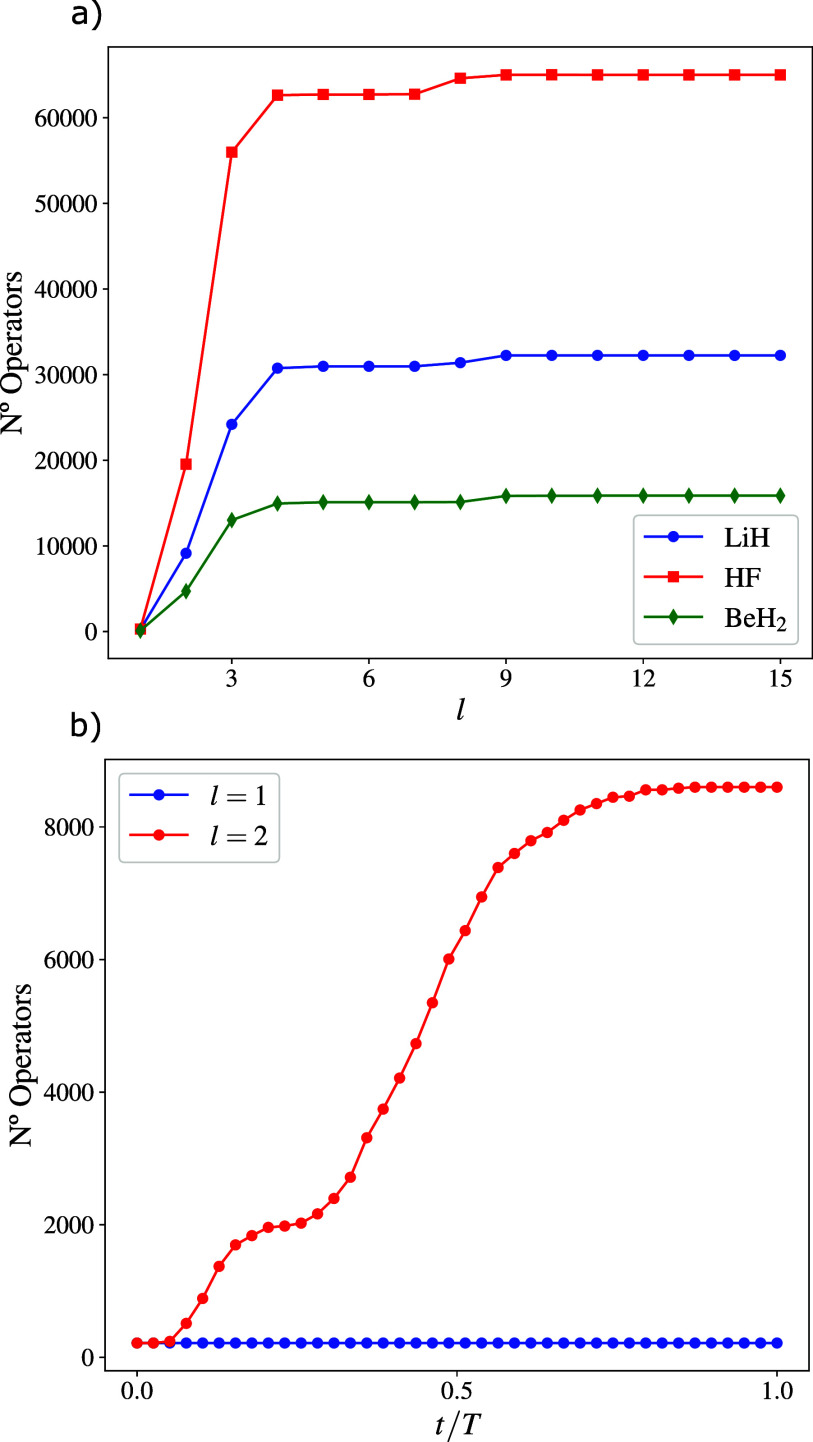
Number of operators in the operator pool {*Ĝ*_*j*_}_*j* = 1_^η^ for: (a) LiH, HF and BeH_2_ in function of *l*-th order of approximation of AGP and: (b) for LiH with time-dependent approximation for *l* = 1, 2.

It would be desirable to obtain an intermediate number of operators between different *l*-th orders, particularly at low orders where the variation is the most significant. To this end, we propose an approach based on approximations that are induced by the amplitude order of the schedule function λ­(*t*), which, combined with the TPD algorithm, yields different numbers of operators depending on the chosen time using the time-dependent Hamiltonian of eq 15. Recall that λ­(*t*) is time dependent, with λ(0) = 0 at the initial time, λ­(*T*) = 1 at the final time, and λ̇(0) = λ̇(*T*) = 0. The schedule function used in this work is

$$\lambda (t)=\sin^{2}\;(\frac{\pi }{2}\sin^{2}\left(\frac{\pi t}{2T}\right))$$ 23

We use the nested commutator expressions as functions of time, but retaining only the dependence on λ­(*t*), without including α_*k*_(*t*) or λ̇(*t*). This choice is justified because λ̇(*t*) multiplies all commutators uniformly and therefore does not affect the number of operators, while α_*k*_(*t*) is obtained by minimizing the action *S*_*l*_ and can be absorbed into the variational parameter to be optimized. For example, for *l* = 2, the time-dependent function that defines the operator pool is

$${\hat{G}}_{j}^{t\prime }\in \{[{\hat{H}}_{i},{\hat{H}}_{f}],(1-\lambda^{2}(t^{\prime }))[{\hat{H}}_{i},[{\hat{H}}_{i},[{\hat{H}}_{i},{\hat{H}}_{f}]]]+\lambda (t^{\prime })(1-\lambda (t^{\prime }))([{\hat{H}}_{i},[{\hat{H}}_{f},[{\hat{H}}_{i},{\hat{H}}_{f}]]]+[{\hat{H}}_{f},[{\hat{H}}_{i},[{\hat{H}}_{i},{\hat{H}}_{f}]]])+\lambda^{2}(t^{\prime })[{\hat{H}}_{f},[{\hat{H}}_{f},[{\hat{H}}_{i},{\hat{H}}_{f}]]]\}$$ 24

where *t′*∈ [0, *T*] is the chosen time. For different values of *t′*, the number of operators in the pool changes because the TPD algorithm discards Pauli strings whose coefficients fall below a given threshold, and λ­(*t′*) modulates these coefficients. It is important to note that this method for operator pool makes use of the TPD algorithm, which relies on the matrix representation of the commutators and is therefore not scalable with the number of qubits. Nevertheless, our algorithm does not depend on TPD algorithm, which is employed here solely for numerical analysis. Other criteria, as proposed in refs 37,38 , may also be used to obtain an intermediate operator pool size between *l* = 1 and *l* = 2 without matrix representation.

To illustrate this approach, Figure 1b shows that for *l* = 2 and the LiH molecule the number of operators varies as a function of time. This behavior can be exploited for different *l*-th orders, providing a way to obtain intermediate operator pool size between *l* = 1 and higher *l*-th orders. We examine how this approach for operator pool influences the performance of the CD-ADAPT algorithm in the following section.

### 3.2 Numerical Results with CD-ADAPT Algorithm

Numerical simulations were performed using the Qiskit SDK^39^ to implement the TPD algorithm for operator pool generation for *l* = 1 and *l* = 2, and to execute the CD-ADAPT algorithm. The Qiskit Nature library^40^ was employed to construct the molecular Hamiltonians *Ĥ*_f_ of the form of eq 5 via the built-in PySCF driver and the mappers provided. The performance of the CD-ADAPT algorithm was evaluated for the LiH, HF, and BeH_2_ molecules. For the LiH and BeH_2_ the electronic structure Hamiltonian was initialized across a range of interatomic distances. We employed an *Active Space Transformation* consisting of 4 electrons and 5 spatial orbitals resulting in encoding 10 spin–orbitals in 10 qubits, using the STO-3G basis set and the Jordan-Wigner mapping. For the HF molecule, the same active space parameters were applied, but utilizing the larger 6–31G basis set. Optimization within the VQE subroutines was performed using the L-BFGS-B optimizer, with the gradient convergence threshold set to ϵ = 10^–2^.

As discussed in the previous section, the number of operators generated by the *l*-th order approximate AGP is inherently dependent on the approximation order *l* and the specific time *t′* at which the AGP is evaluated if the TDP algorithm is used to interpolate between different orders of *l*. We conducted a comparison to determine how these parameters impact in the precision in recovering the ground-state energy. Specifically, we analyzed the convergence behavior for *l* = {1, 2} and, for *l* = 2, compared time points *t′* = {0.25, 0.75}. The results of these simulations are illustrated in Figures 2–4.

**Figure 2:**
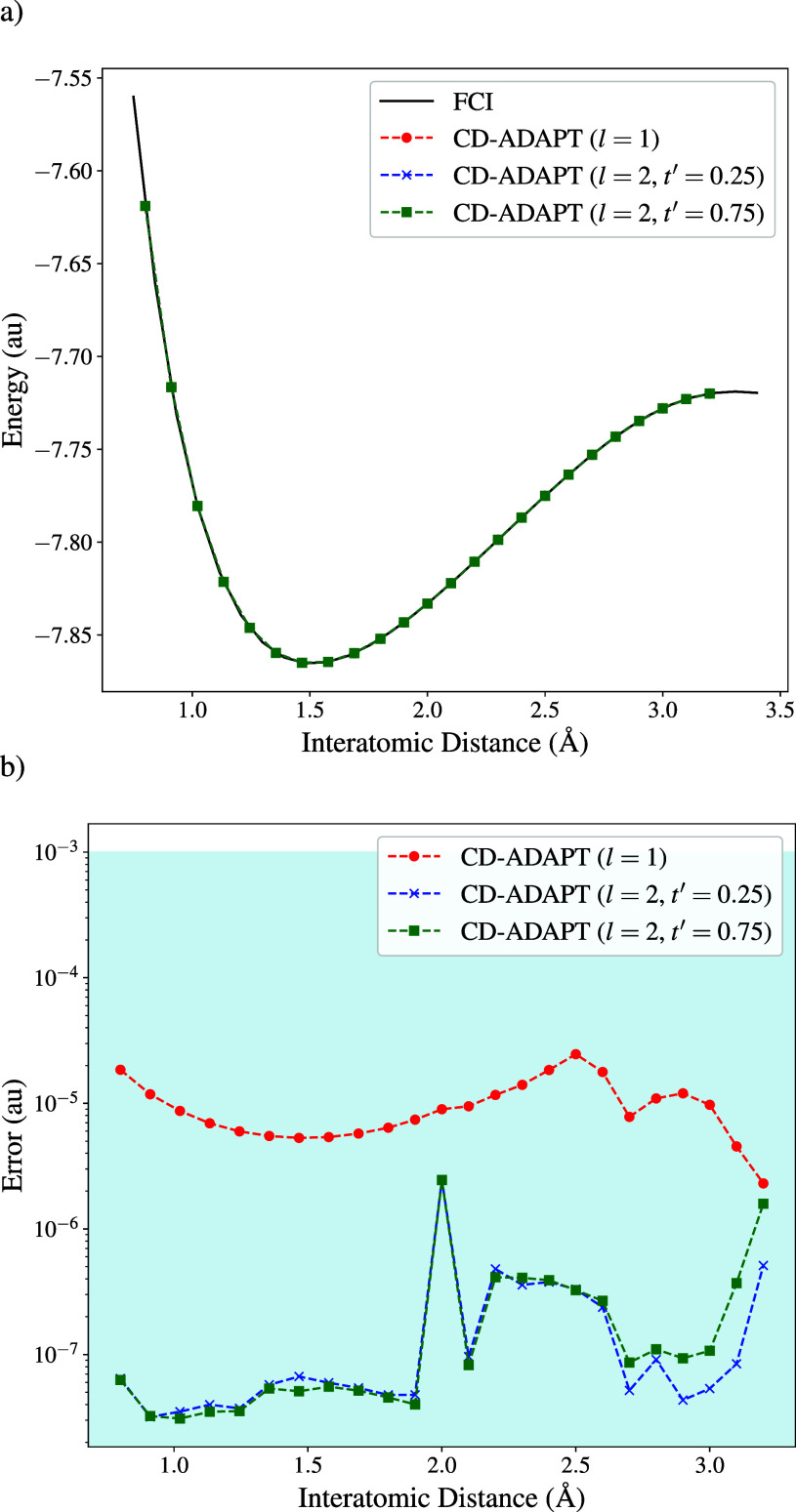
Ground state energy and corresponding error for LiH calculated via CD-ADAPT. (a) Energy dissociation curve compared to the FCI. (b) Absolute error as a function of interatomic distance for first (*l* = 1) and second-order (*l* = 2) approximations with time parameters *t′* = 0.25 and *t′* = 0.75. The blue shaded region indicates the range within chemical accuracy.

The performance of the CD-ADAPT ansatz for the LiH molecule was evaluated by analyzing the ground state energy surface and the corresponding absolute errors relative to the Full Configuration Interaction (FCI) benchmark (see Figure 2). Regarding the expansion order *l*, Figure 2a shows that the first-order approximation (*l* = 1) successfully captures the qualitative dissociation profile. The error analysis in Figure 2b reveals that *l* = 1 yields errors in the order of 10^–5^ Ha. Increasing the order to *l* = 2 results in a significant accuracy improvement, reducing errors by approximately up to 2 orders of magnitude (down to 10^–5^–10^–7^ Ha), highlighting that generating the operator pool from higher-order counterdiabatic terms yields more expressive operators.

The CD-ADAPT algorithm was further tested on the HF molecule, a system exhibiting significant electron correlation effects driven by the strong polarity induced by the fluorine atom. The computed energy landscape and the corresponding absolute error profiles relative to the exact FCI benchmark are presented in Figure 3. Regarding the expansion order, the data in Figure 3a confirms that *l* = 1 is sufficient to reproduce the behavior of the dissociation curve under chemical accuracy, yielding errors consistently around 10^–5^ Ha (see Figure 3b). Conversely, the inclusion of *l* = 2 in the operator pool yields a drastic reduction in the error. Notably, the error drops by several orders of magnitude, reaching the 10^–6^–10^–8^ Ha. This behavior reinforces the notion that the operator pool derived from higher-order nested commutators is more effective in expressing unitary operators that approximates the HF ground state.

**Figure 3:**
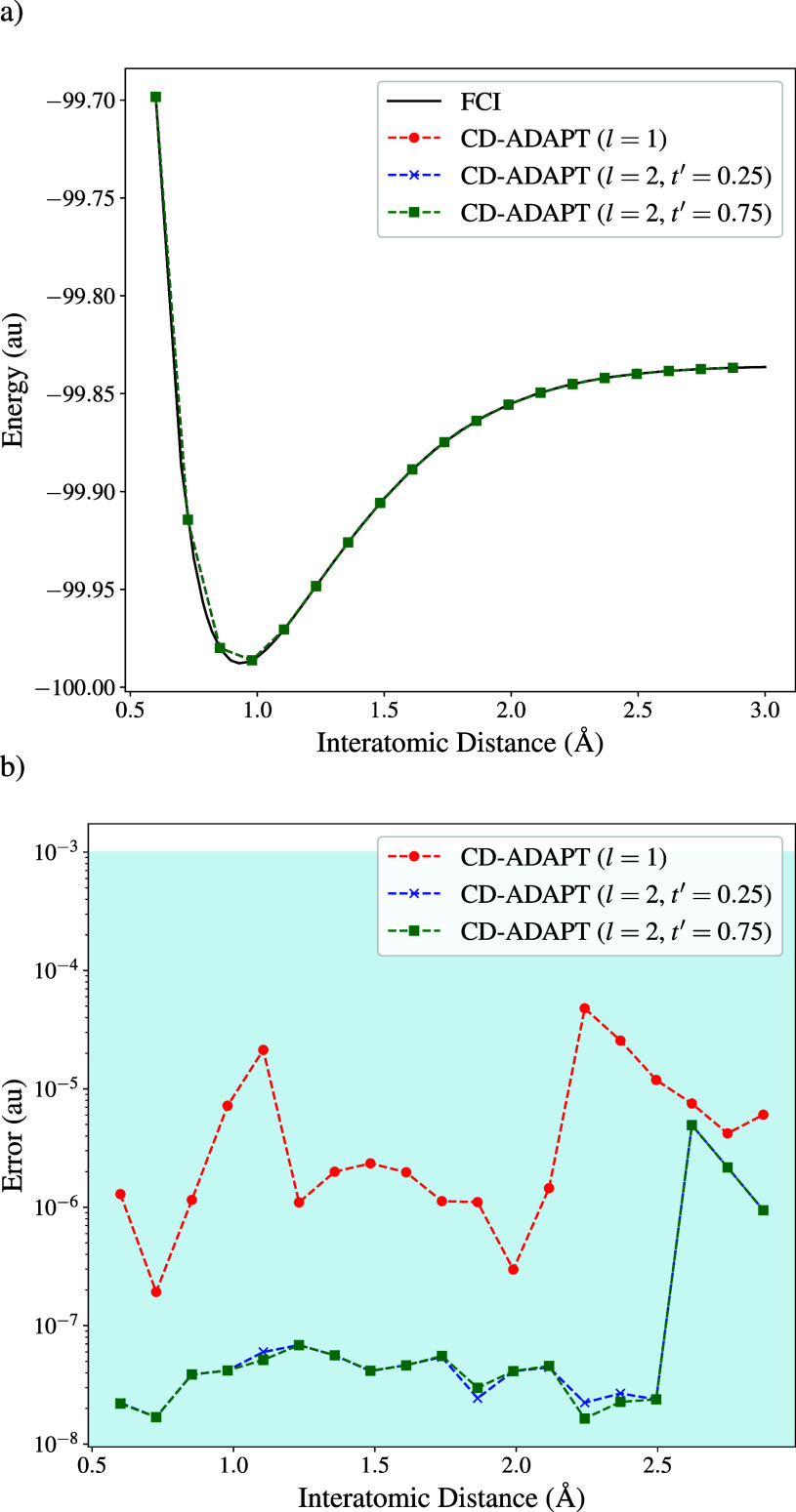
Ground state energy and corresponding error for HF calculated via CD-ADAPT. (a) Energy dissociation curve compared to the FCI. (b) Absolute error as a function of interatomic distance for first (*l* = 1) and second-order (*l* = 2) approximations with time parameters *t′* = 0.25 and *t′* = 0.75. The blue shaded region indicates the range within chemical accuracy.

Finally, we tested our algorithm on the BeH_2_ molecule. Consistent with the previous results, as shown in Figure 4, using the operator pool with *l* = 1 yields errors below chemical accuracy and on the order of 10^–5^ Ha. In this case, the *l* = 2 operator pool exhibits significantly better performance than *l* = 1, while showing similar performance for *t′* = 0.25 and *t′* = 0.75 across all considered bond distances, with errors on the order of 10^–7^–10^–8^ Ha. This shows that using more operators from the *l* = 2 approximation does not always guarantee better accuracy, and that employing a reduced subset of these operators can already lead to a significant improvement compared to the *l* = 1 case. Future work may focus on developing improved criteria for selecting operators from the *l* = 2 or higher-order approximations of the AGP, with the aim of constructing a more compact operator pool.

**Figure 4:**
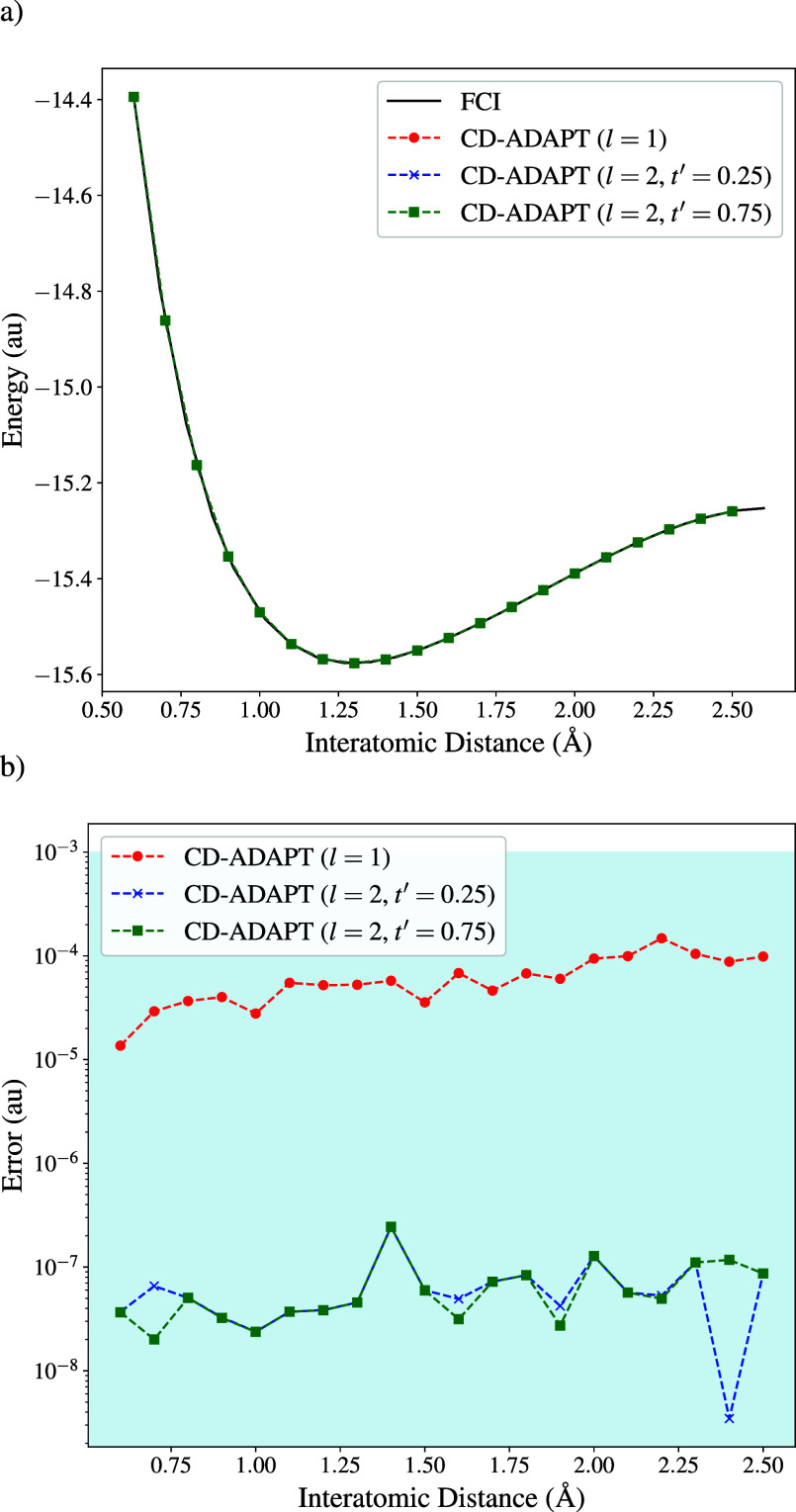
Ground state energy and corresponding error for BeH_2_ calculated via CD-ADAPT. (a) Energy dissociation curve compared to the FCI. (b) Absolute error as a function of interatomic distance for first (*l* = 1) and second-order (*l* = 2) approximations with time parameters *t′* = 0.25 and *t′* = 0.75. The blue shaded region indicates the range within chemical accuracy.

### 3.3 Comparison with Digitized Counterdiabatic Quantum Optimization and Fermionic ADAPT-VQE

To benchmark the performance of the proposed CD-ADAPT algorithm 1, we compared it against the two methods that inspired its development: the DCQO and the Fermionic ADAPT-VQE. The DCQO was implemented using Qiskit by performing a Trotterization of the unitary evolution operator defined in eq 17, with a fixed total evolution time *T* = 1 and *N*_*t*_ = 2 Trotter steps. The Fermionic ADAPT-VQE was implemented utilizing the built-in solvers provided by Qiskit Nature^40^ and Qiskit Algorithms.

We analyzed the ground state accuracy relative to the FCI benchmark achieved by these algorithms across various interatomic distance intervals. Figure 5 shows that CD-ADAPT consistently achieves higher accuracy than the other algorithms considered, more precisely, with respect to the ADAPT-VQE algorithm, the *l* = 1 instance of the CD-ADAPT achieves 1 order of magnitude improvement in the estimation of the ground state energy, while for the *l* = 2 instance and *t′* = 0.75 shows an improvement of 3 orders of magnitude. With respect to the DCQO, this improvements scales up to three and 5 orders of magnitude, respectively. We also observe that the DCQO remains above chemical accuracy for *N*_*t*_ = 2, indicating a poor approximation of the counterdiabatic evolution with the selected numerical parameters. This value of *N*_*t*_ was intentionally chosen to analyze the number of controlled-NOT (CNOT) gates, revealing that DCQO is already less efficient than our approach in terms of circuit complexity. Although increasing *N*_*t*_ would improve the accuracy, it is done at the cost of a significantly larger number of CNOT, rendering the method impractical. Furthermore, we evaluate the circuit complexity by quantifying the number of CNOT gates required to implement each ansatz at a fixed interatomic distance for each molecule. Circuit transpilation was performed using the transpile subroutine in Qiskit with optimization level 3 on the GenericBackendV2. The comparative results are detailed in Table 2 for BeH_2_ (*r* = 1.50 Å), Table 3 for LiH (*r* = 1.58 Å), and Table 4 for HF (*r* = 0.917 Å).

**Table 2:** Comparison of the Error, Number of Variational Parameters, and CNOT Gates for Different Algorithms Applied to BeH_2_ at Distance *r* = 1.500 Å^a^

algorithm	error (a.u.)	*N*° parameters	*N*° CNOTs
CD-ADAPT (*l* = 1)	3.56 × 10^–5^	18	208
CD-ADAPT (*l* = 2, *t′* = 0.25)	5.98 × 10^–8^	27	318
CD-ADAPT (*l* = 2, *t′* = 0.75)	5.98 × 10^–8^	27	324
ADAPT-VQE	3.07 × 10^–4^	6	419
DCQO (*l* = 1)	1.17 × 10^–2^		1359
DCQO (*l* = 2)	8.72 × 10^–3^		5665

a For all ADAPT-based algorithms, the threshold is fixed at ϵ = 10^–2^, while for DCQO, the number of Trotter steps is set to two.

**Figure 5:**
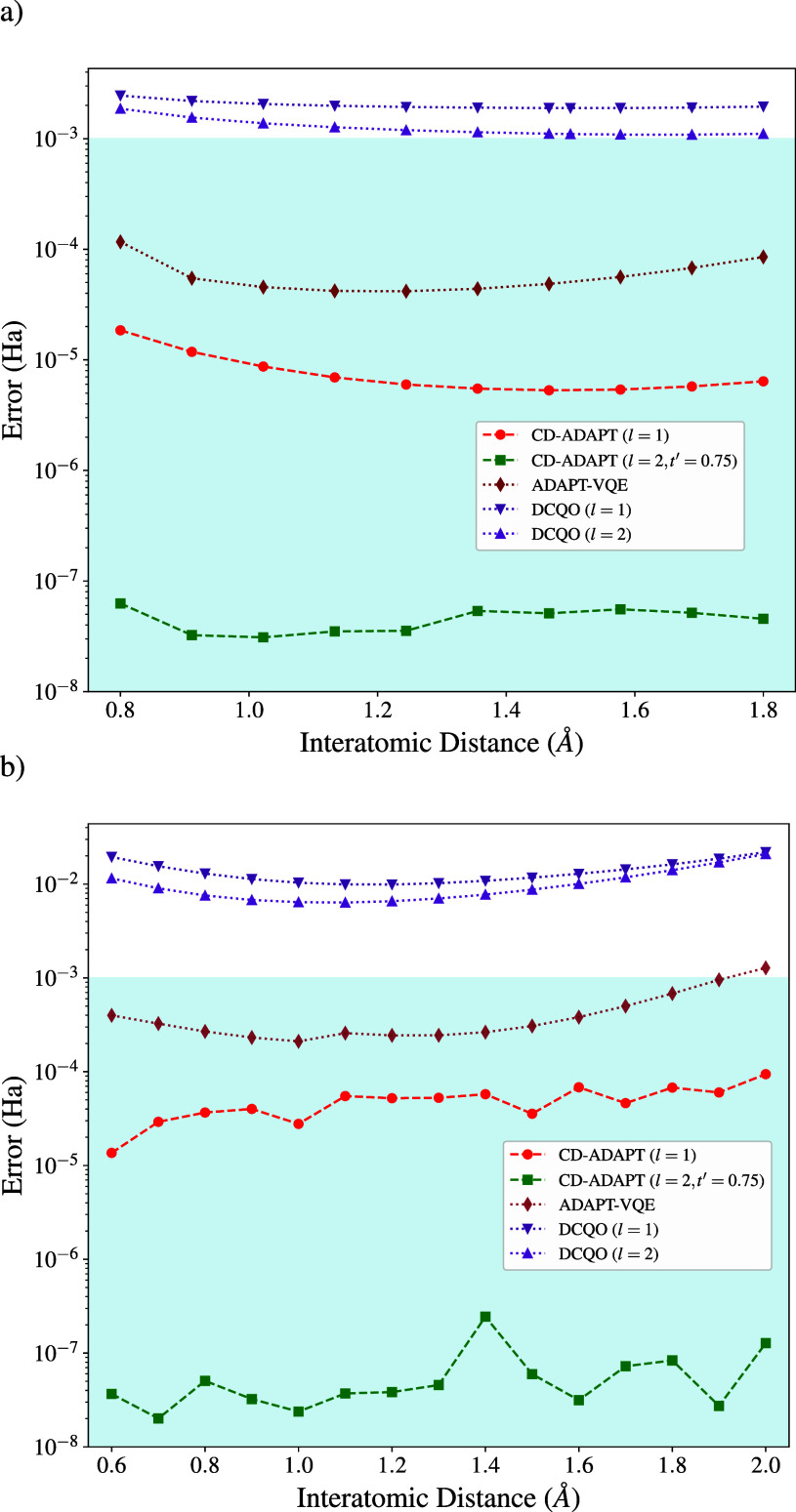
Absolute error calculated for CD-ADAPT, ADAPT-VQE, and DCQO vs interatomic distance. (a) For LiH and (b) for BeH_2_. The blue shaded region indicates the range within chemical accuracy.

**Table 3:** Comparison of the Error, Number of Variational Parameters, and CNOT Gates for Different Algorithms Applied to LiH at Distance *r* = 1.578 Å^a^

algorithm	error (a.u.)	*N*° parameters	*N*° CNOTs
CD-ADAPT (*l* = 1)	5.39 × 10^–6^	12	134
CD-ADAPT (*l* = 2, *t′* = 0.25)	5.96 × 10^–8^	32	368
CD-ADAPT (*l* = 2, *t′* = 0.75)	5.55 × 10^–8^	31	376
ADAPT-VQE	5.61 × 10^–5^	10	884
DCQO (*l* = 1)	1.89 × 10^–3^		1415
DCQO (*l* = 2)	1.09 × 10^–3^		5269

a For all ADAPT-based algorithms, the threshold is fixed at ϵ = 10^–2^, while for DCQO, the number of Trotter steps is set to two.

**Table 4:** Comparison of the Error, Number of Variational Parameters, and CNOT Gates for Different Algorithms Applied to HF at Distance *r* = 0.917 Å^a^

algorithm	error (a.u.)	*N*° parameters	*N*° CNOTs
CD-ADAPT (*l* = 1)	8.19 × 10^–6^	14	156
CD-ADAPT (*l* = 2, *t′* = 0.25)	1.39 × 10^–6^	25	274
CD-ADAPT (*l* = 2, *t′* = 0.75)	7.82 × 10^–8^	36	408
ADAPT-VQE	1.82 × 10^–5^	12	993
DCQO (*l* = 1)	2.66 × 10^–2^		2343
DCQO (*l* = 2)	5.58 × 10^–3^		14,083

a For all ADAPT-based algorithms, the threshold is fixed at ϵ = 10^–2^, while for DCQO, the number of Trotter steps is set to two.

For the comparison with ADAPT-VQE, another important aspect is the choice of optimizer used at each VQE step. To assess the robustness of the comparison, we considered different optimizers and found that our algorithm consistently performs better than standard ADAPT-VQE. As shown in Tables 5 and 6, the final error remains within the same order of magnitude across the optimizers considered. In all cases, CD-ADAPT with *l* = 1 yields a lower error than ADAPT-VQE.

**Table 5:** Comparison of the Error between CD-ADAPT and ADAPT-VQE with Different Optimizers for BeH_2_ at Distance *r* = 1.500 Å

algorithm	optimizer	error (a.u.)
CD-ADAPT (*l* = 1)	L-BFGS-B	3.54 × 10^–5^
CD-ADAPT (*l* = 1)	COBYLA	1.97 × 10^–5^
CD-ADAPT (*l* = 1)	SLSQP	5.12 × 10^–5^
ADAPT-VQE	L-BFGS-B	4.85 × 10^–4^
ADAPT-VQE	COBYLA	4.85 × 10^–4^
ADAPT-VQE	SLSQP	4.86 × 10^–4^

**Table 6:** Comparison of the Error between CD-ADAPT and ADAPT-VQE with Different Optimizers for HF at Distance *r* = 0.917 Å

algorithm	optimizer	error (a.u.)
CD-ADAPT (*l* = 1)	L-BFGS-B	8.19 × 10^–6^
CD-ADAPT (*l* = 1)	COBYLA	8.22 × 10^–6^
CD-ADAPT (*l* = 1)	SLSQP	8.21 × 10^–6^
ADAPT-VQE	L-BFGS-B	1.85 × 10^–5^
ADAPT-VQE	COBYLA	2.13 × 10^–5^
ADAPT-VQE	SLSQP	1.86 × 10^–5^

The results demonstrate that, for the given gradient threshold, our CD-ADAPT approach significantly outperforms the benchmark methods in terms of both accuracy and circuit efficiency (CNOT count). As shown at Tables 2–4, the CD-ADAPT algorithm requires ∼100–500 fewer CNOT gates than ADAPT-VQE for this specific configuration. Although ADAPT-VQE generally employs fewer variational parameters, this comes at the cost of lower accuracy for the same convergence threshold. In contrast, CD-ADAPT constructs a pool of operators derived from the counterdiabatic expansion, enabling deeper convergence with more compact circuits.

### 3.4 Comparison with Alternative ADAPT Variants

In recent years, a variety of alternative versions of ADAPT-VQE have been proposed. In particular, a notable example is Qubit-ADAPT-VQE,^17^ which constructs the operator pool with individual Pauli strings, similarly to our approach. However, there is a key distinction between both constructions. In Qubit-ADAPT-VQE, the Pauli strings originate from Fermionic excitations operators mapped onto qubit operators, and therefore retain the underlying structure of excitations between occupied and unoccupied orbitals respect to reference state. In contrast, in our approach there is no explicit occupied-unoccupied orbital structure, since the operators are generated directly from the molecular Hamiltonian mapped to qubit operators, *Ĥ*_f_ and the commutators with *Ĥ*_*i*_. As a result, the pool is not constrained by restriction in orbital excitations, but instead reflects the operator content arising from the Hamiltonian itself. Generalized singles and doubles excitations are also considered in Qubit-ADAPT-VQE, where all combinations of excitations between orbitals are included. In this case, the terms *â*_*j*_^†^ *â*_*i*_ and *â*_*l*_^†^ *â*_*k*_^†^ *â*_*j*_ *â*_*i*_ in *Ĥ*_f_ correspond to terms of the excitations considered in these approaches and thus form a subset of them. Consequently, after taking commutators in the approximate AGP, different Pauli strings can emerge in our algorithm compared to Qubit-ADAPT-VQE.

In the line of excitation-based operator pools, a more recent development is the coupled exchange operators ADAPT-VQE (CEO-ADAPT-VQE).^19^ In this approach, instead of Fermionic excitations, qubit excitations are considered directly, thus avoiding the need for a Fermion-to-qubit mapping in the construction of the operator pool. Moreover, CEO-ADAPT-VQE introduces linear combinations of qubit excitations acting on the same set of spin–orbitals, which have been shown to provide notorious advantages in terms of CNOT count and circuit depth compared to previous ADAPT-VQE variants. Similar to generalized single and double excitations between orbitals, CEO-ADAPT-VQE does not impose an occupied–unoccupied orbital restriction, and qubit excitations are defined over arbitrary sets of qubits.

Therefore, it is of interest to compare these generalized excitation-based pools with our AGP-based pool, since the underlying logic is fundamentally different. We implement these algorithms (referred to as Qubit-ADAPT and CEO-ADAPT) using the authors’ GitHub code,^41^ which employs OpenFermion^42^ to define the molecular Hamiltonian on top of which we perform an *Active Space Transformation* with 4 electrons and 5 spatial orbitals, corresponding to 10 spin–orbitals encoded in 10 qubits, while Qiskit is used for the quantum circuit implementation, consistent with the setup used in our algorithm.

An important aspect when comparing pools of different sizes is that the $l_{2}$ -norm of the gradient vector can be inflated for larger pools, potentially delaying convergence. Therefore, we consider three threshold values and report the absolute error as a function of the ADAPT iterations until convergence. In our case, CD-ADAPT depends on the *l*-th order approximation of the AGP, which leads to operator pools of increasing size as *l* increases. This growth can significantly affect both the gradient norm and the convergence behavior, making direct comparisons across different *l* values nontrivial. To ensure a fair comparison, we run the *l* = 1 pool until convergence and then evaluate the *l* = 2 pool at a fixed iteration corresponding to the point at which *l* = 1 reaches convergence. In this way, we can see the effect of the higher-order AGP terms without introducing biases due to differences in pool size or convergence rates.

In Figure 6a, we show the absolute error as a function of ADAPT iterations until convergence (except for CD-ADAPT with *l* = 2, which is evaluated at a fixed iteration) for a threshold ϵ = 10^–1^. All algorithms reach chemical accuracy and exhibit very similar energies within the first four iterations. Qubit-ADAPT requires more iterations to converge, consistent with the larger pool size and the corresponding inflation of the gradient $l_{2}$ -norm. Figure 6b presents the same analysis for ϵ = 10^–2^. In this case, a clear difference emerges between CD-ADAPT with *l* = 1 and *l* = 2, where distinct operators from the pool are selected, highlighting the importance of a more accurate AGP approximation. Qubit-ADAPT shows a similar convergence trend to CD-ADAPT with *l* = 2, while CEO-ADAPT reaches convergence in only seven iterations, achieving an absolute error comparable to CD-ADAPT with *l* = 2. In Figure 7, for ϵ = 10^–3^, CD-ADAPT with *l* = 2 consistently achieves lower errors than both *l* = 1 and Qubit-ADAPT. Additionally, CEO-ADAPT converges more rapidly than all other variants. It is important to mention that CEO-ADAPT contains considerably fewer CNOTs than the other algorithms (Table 7). This shows that considering linear combinations of excitations improves the circuit depth and constitutes an efficient strategy to reduce the number of iterations. As future work, we plan to explore a similar strategy for our algorithm, where linear combinations of commuting operators, or operators grouped according to other suitable criteria, are assigned a single variational parameter. In addition, alternative approximations to the AGP could be considered in order to study how they modify the structure of the operator pool. We consider these directions promising topics for future work.

**Figure 6:**
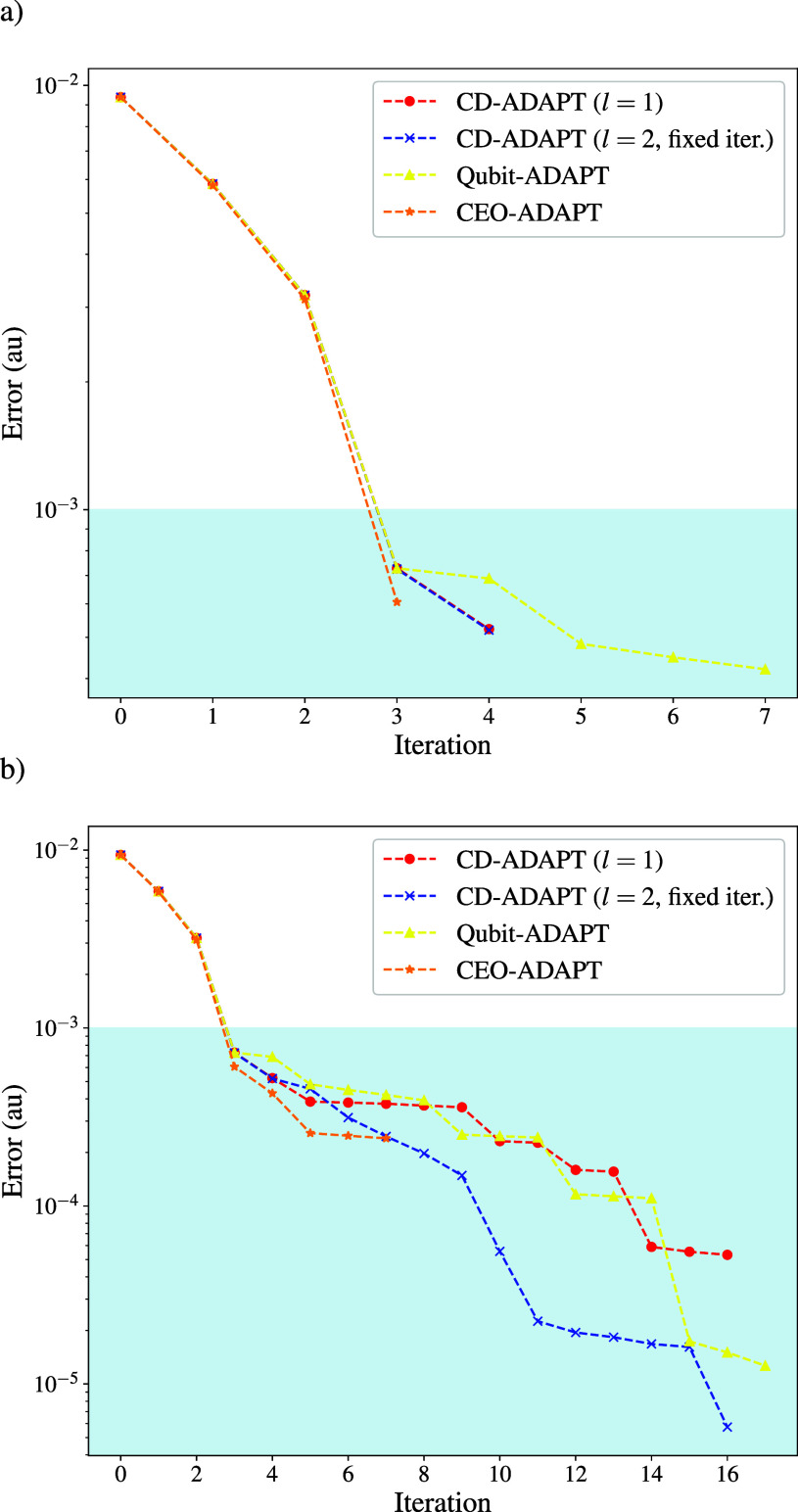
Absolute error as a function of ADAPT iterations for the BeH_2_ molecule at *r* = 1.326 Å using CD-ADAPT with *l* = 1 and *l* = 2 (with *t′* = 0.25), where the *l* = 2 results are evaluated at a fixed iteration corresponding to the convergence point of *l* = 1. Results for Qubit-ADAPT with generalized single and double excitations and CEO-ADAPT with generalized qubit excitations are also shown. (a) Threshold ϵ = 10^–1^ and (b) threshold ϵ = 10^–2^.

**Figure 7:**
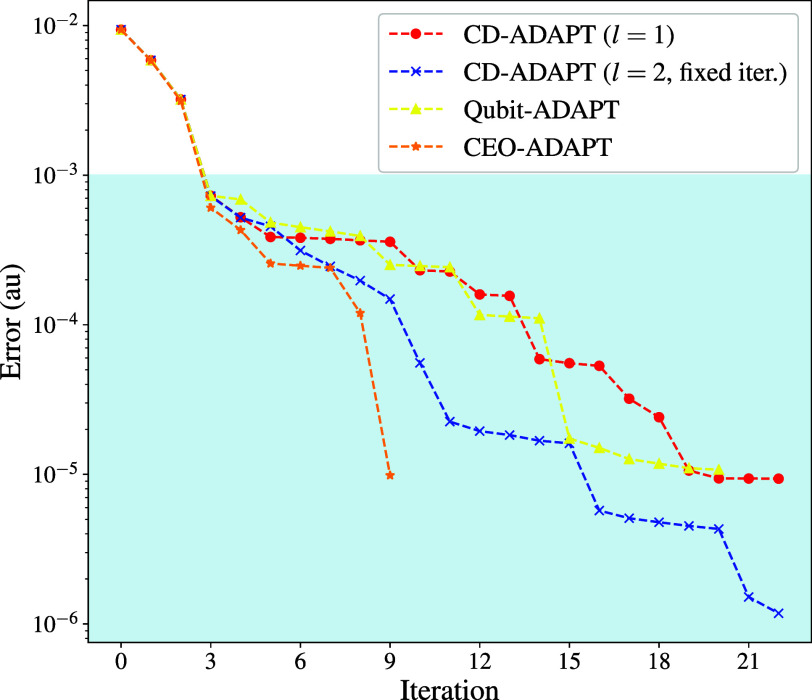
Absolute error as a function of ADAPT iterations for the BeH_2_ molecule at *r* = 1.326 Å using CD-ADAPT with *l* = 1 and *l* = 2 (with *t′* = 0.25), where the *l* = 2 results are evaluated at a fixed iteration corresponding to the convergence point of *l* = 1. Results for Qubit-ADAPT with generalized single and double excitations and CEO-ADAPT with generalized qubit excitations are also shown for threshold ϵ = 10^–3^.

**Table 7:** Comparison of the Pool Size, Error, and CNOT Gates for Different ADAPT Variants, for BeH_2_ at Distance *r* = 1.326 for the Threshold ϵ = 10^–3^

algorithm	pool size	error (a.u.)	*N*° CNOTs
CD-ADAPT (*l* = 1)	108	9.35 × 10^–6^	225
CD-ADAPT (*l* = 2, fixed-it)	968	1.18 × 10^–6^	241
Qubit-ADAPT	1240	1.07 × 10^–5^	234
CEO-ADAPT	510	9.85 × 10^–6^	152

## 4 Conclusion

We proposed a hybrid quantum–classical algorithm that constructs an operator pool from the counterdiabatic protocol using approximate adiabatic gauge potential and combines it with the ADAPT-VQE^16^ strategy to build the ansatz. This approach enabled the inclusion of higher order of approximate adiabatic gauge potential without a corresponding increase in the ansatz circuit depth as observed in conventional DCQO,^24^ since the ADAPT-VQE gradient criterion selects only the more relevants operators for approaching to ground state. Numerical simulations show that our algorithm achieves a lower number of CNOT gates and an error reduced by approximately 5 orders of magnitude compared to DCQO with a Trotter step number equal to two. In addition, the operator pool obtained from the counterdiabatic protocol appeared to be more efficient than that constructed from Fermionic singles and doubles excitations. This becomes evident when comparing with the Fermionic ADAPT-VQE algorithm, where our approach achieves a lower number of CNOT gates and an error that is 3 orders of magnitude smaller. These results indicated that accelerated adiabatic dynamics assisted by counterdiabatic driving can provide a more effective route to the ground state of molecular Hamiltonian than operator selections based solely on physically motivated interaction terms, such as single and double excitations.

To follow the counterdiabatic protocol, we proposed an initial Hamiltonian whose ground state is the Hartree–Fock state and which is composed of local operators. This choice requires prior knowledge of the Hartree–Fock state, which is typically known and used as the reference state in molecular simulation algorithms. In addition, we employed the TPD algorithm to obtain the Pauli strings associated with the approximate adiabatic gauge potential at orders *l* = 1 and *l* = 2. For *l* = 2, a fixed time dependence was considered, where the selected times are *t′* = 0.25 and *t′* = 0.75, allowing us to generate operator pools with intermediate sizes between the *l* = 1 and *l* = 2. The numerical simulations of our algorithm were performed for the LiH, HF, and BeH_2_ molecules, yielding results below chemical accuracy when using the operator pool with *l* = 1, and achieving an improvement of approximately 2 orders of magnitude when increasing the operator pool to *l* = 2.

Finally, we compare our algorithm with state-of-the-art variants of ADAPT-VQE, such as Qubit-ADAPT-VQE and CEO-ADAPT-VQE. We observe comparable results between CD-ADAPT with *l* = 1 and Qubit-ADAPT-VQE, while improved performance is obtained for *l* = 2. CEO-ADAPT-VQE remains the most efficient in terms of convergence, with fewer iterations and lower CNOT counts than our algorithm. It is important to note that CEO-ADAPT-VQE, while highly effective, is specifically tailored to molecular systems through its operator pool. In contrast, our approach provides a more transferable strategy that can be extended to other contexts, such as quantum simulations and combinatorial optimization problems, as the pool is constructed based on counterdiabatic driving within an adiabatic framework. This highlights the potential of physics-inspired operator pools.

Overall, these results demonstrate that our approach achieves competitive performance in the context of NISQ and early fault-tolerant quantum computing for molecular simulation.

## Data Availability

The data generated with the code that support the findings of this study are available publicly in this github repository: https://github.com/jervaal/cd-adapt-vqe.

## References

[1] Arute F. (2019). Quantum supremacy
using a programmable superconducting processor. Nature.

[2] Zhong H.-S. (2021). Phase-programmable
gaussian boson sampling using stimulated squeezed light. Phys. Rev. Lett..

[3] Wu Y. (2021). Strong Quantum
Computational Advantage Using a Superconducting Quantum Processor. Phys. Rev. Lett..

[4] Madsen L. (2022). Quantum computational
advantage with a programmable photonic processor. Nature.

[5] Kim Y., Eddins A., Anand S., Wei K. X., van den
Berg E., Rosenblatt S., Nayfeh H., Wu Y., Zaletel M., Temme K., Kandala A. (2023). Evidence for the utility of quantum
computing before fault tolerance. Nature.

[6] Zhu Q. (2022). Quantum computational
advantage via 60-qubit 24-cycle random circuit sampling. Sci. Bull..

[7] Morvan A. (2024). Phase transitions
in random circuit sampling. Nature.

[8] Acharya R. (2024). Quantum error
correction below the surface code threshold. Nature.

[9] Gao D. (2025). Establishing
a New Benchmark in Quantum Computational Advantage with 105-qubit
Zuchongzhi 3.0 Processor. Phys. Rev. Lett..

[10] Bharti K. (2022). Noisy intermediate-scale
quantum algorithms. Rev. Mod. Phys..

[11] Peruzzo A., McClean J., Shadbolt P., Yung M.-H., Zhou X.-Q., Love P. J., Aspuru-Guzik A., O’Brien J. L. (2014). A variational
eigenvalue solver on a photonic quantum processor. Nat. Commun..

[12] Kandala A., Mezzacapo A., Temme K., Takita M., Brink M., Chow J. M., Gambetta J. M. (2017). Hardware-efficient variational quantum
eigensolver for small molecules and quantum magnets. Nature.

[13] Fontana E., Herman D., Chakrabarti S., Kumar N., Yalovetzky R., Heredge J., Hari Sureshbabu S., Pistoia M. (2024). Characterizing barren
plateaus in quantum ansätze with the adjoint representation. Nat. Commun..

[14] Ragone M., Bakalov B. N., Sauvage F., Kemper A. F., Ortiz
Marrero C., Larocca M., Cerezo M. (2024). A Lie algebraic theory
of barren plateaus for deep parameterized quantum circuits. Nat. Commun..

[15] Grimsley H. R., Barron G. S., Barnes E., Economou S. E., Mayhall N. J. (2023). Adaptive,
problem-tailored variational quantum eigensolver mitigates rough parameter
landscapes and barren plateaus. npj Quantum
Inf..

[16] Grimsley H. R., Economou S. E., Barnes E., Mayhall N. J. (2019). An adaptive
variational
algorithm for exact molecular simulations on a quantum computer. Nat. Commun..

[17] Tang H. L., Shkolnikov V., Barron G., Grimsley H., Mayhall N., Barnes E., Economou S. E. (2021). Qubit-ADAPT-VQE: An Adaptive Algorithm
for Constructing Hardware-Efficient Ansätze on a Quantum Processor,
PRX. Quantum.

[18] Yordanov Y. S., Armaos V., Barnes C. H. W., Arvidsson-Shukur D. R. M. (2021). Qubit-excitation-based
adaptive variational quantum eigensolver. Commun.
Phys..

[19] Ramôa M., Anastasiou P. G., Santos L. P., Mayhall N. J., Barnes E., Economou S. E. (2025). Reducing the resources required by
ADAPT-VQE using
coupled exchange operators and improved subroutines. npj Quantum Inf..

[20] Farrell R. C., Illa M., Ciavarella A. N., Savage M. J. (2024). Scalable Circuits
for Preparing Ground States on Digital Quantum Computers: The Schwinger
Model Vacuum on 100 Qubits. PRX Quantum.

[21] Yao Y.-X., Gomes N., Zhang F., Wang C.-Z., Ho K.-M., Iadecola T., Orth P. P. (2021). Adaptive
Variational Quantum Dynamics
Simulations. PRX Quantum.

[22] Anastasiou P. G., Chen Y., Mayhall N. J., Barnes E., Economou S. E. (2024). TETRIS-ADAPT-VQE:
An adaptive algorithm that yields shallower, denser circuit Ansätze. Phys. Rev. Res..

[23] Vaquero-Sabater N., Carreras A., Casanova D. (2025). Pruned-ADAPT-VQE:
Compacting Molecular
Ansätze by Removing Irrelevant Operators. J. Chem. Theory Comput..

[24] Hegade N. N., Chen X., Solano E. (2022). Digitized counterdiabatic
quantum
optimization. Phys. Rev. Res..

[25] Farhi E., Goldstone J., Gutmann S., Lapan J., Lundgren A., Preda D. (2001). A Quantum
Adiabatic Evolution Algorithm Applied to Random Instances
of an NP-Complete Problem. Science.

[26] Chandarana P., Hegade N. N., Montalban I., Solano E., Chen X. (2023). Digitized
counterdiabatic quantum algorithm for protein folding. Phys. Rev. Appl..

[27] Dalal A. (2024). Digitized counterdiabatic
quantum algorithms for logistics scheduling. Phys. Rev. Appl..

[28] Cadavid A. G., Montalban I., Dalal A., Solano E., Hegade N. N. (2024). Efficient
digitized counterdiabatic quantum optimization algorithm within the
impulse regime for portfolio optimization. Phys.
Rev. Appl..

[29] Ferreiro-Vélez, J. ; Iriarte-Zendoia, I. ; Ban, Y. ; Chen, X. , Shortcuts for Adiabatic and Variational Algorithms in Molecular Simulation. arXiv:2407.20957 2024, 10.48550/arXiv.2407.20957.

[30] McArdle S. (2020). Quantum computational
chemistry. Rev. Mod. Phys..

[31] Seeley J. T., Richard M. J., Love P. J. (2012). The Bravyi-Kitaev
transformation
for quantum computation of electronic structure. J. Chem. Phys..

[32] Claeys P. W., Pandey M., Sels D., Polkovnikov A. (2019). Floquet-Engineering
Counterdiabatic Protocols in Quantum Many-Body Systems. Phys. Rev. Lett..

[33] Zhu L., Tang H. L., Barron G. S., Calderon-Varg F. A., Mayhall N. J., Barnes E., Economou S. E. (2022). Adaptive
quantum
approximate optimization algorithm for solving combinatorial problems
on a quantum computer. Phys. Rev. Res..

[34] Anastasiou, P. G. ; Mayhall, N. J. ; Barnes, E. ; Economou, S. E. , How to really measure operator gradients in ADAPT-VQE, arXiv:2306.03227 2023, 10.48550/arXiv.2306.03227.

[35] Ramôa, M. , Santos, L. ; Mayhall, N. J. ; Barnes, E. ; Economou, S. E. , Co-Designed Adaptive Quantum State Preparation Protocols. arXiv:2601.20681 2026, 10.48550/arXiv.2601.20681.

[36] Hantzko L., Binkowski L., Gupta S. (2024). Tensorized Pauli decomposition
algorithm. Phys. Scr..

[37] Van
Dyke J. S., Shirali K., Barron G. S., Mayhall N. J., Barnes E., Economou S. E. (2024). Scaling adaptive quantum simulation
algorithms via operator pool tiling. Phys. Rev.
Res..

[38] Long C. K., Dalton K., Barnes C. H. W., Arvidsson-Shukur D. R. M., Mertig N. (2024). Layering and subpool
exploration for adaptive variational
quantum eigensolvers: Reducing circuit depth, runtime, and susceptibility
to noise. Phys. Rev. A.

[39] Javadi-Abhari, A. , Treinish, M. ; Krsulich, K. ; Wood, C. J. ; Lishman, J. ; Gacon, J. ; Quantum computing with Qiskit, arXiv:2405.08810 2024, 10.48550/arXiv.2405.08810.

[40] Qiskit Nature developers and contributors , Qiskit Nature 0.6.0, Zenodo, 2023.

[41] Available at: https://github.com/mafaldaramoa/ceo-adapt-vqe.

[42] McClean J. R. (2020). OpenFermion: The electronic
structure package for quantum computers. Quantum
Sci. Technol..

